# Elucidating the Mechanistic Role of Exogenous Melatonin in Salt Stress Tolerance of Maize (*Zea mays* L.) Seedlings: An Integrated Physiological, Metabolomic, and Proteomic Profiling Analysis

**DOI:** 10.3390/plants14203129

**Published:** 2025-10-10

**Authors:** Zhichao Wang, Linhao Zong, Qiqi Cai, Yinjie Fu, Zhiping Gao, Guoxiang Chen

**Affiliations:** Jiangsu Key Laboratory for Biodiversity and Biotechnology, College of Life Sciences, Nanjing Normal University, 1 Wenyuan Rd., Nanjing 210023, China; w269229@163.com (Z.W.); licht_hao@163.com (L.Z.); c15967768609@163.com (Q.C.); 15158851870@163.com (Y.F.)

**Keywords:** melatonin, salt stress, metabolomics, proteomics, photosynthesis, photosystem II, antioxidant enzymes

## Abstract

Maize (*Zea mays* L.), as a globally significant cereal crop, exhibits high sensitivity to salt stress during early seedling stages. Although melatonin (MT) has demonstrated potential in mitigating abiotic stresses, the specific mechanisms underlying MT-mediated alleviation of salt stress in maize seedlings remain unclear. In this study, we established four treatment groups: control (CK), melatonin treatment (MT), salt stress (NaCl), and combined treatment (NaCl_MT). Metabolomic and proteomic analyses were performed, supplemented by photosynthesis-related experiments as well as antioxidant-related experiments. Metabolomic analysis identified key metabolites in MT-mediated salt stress mitigation. Both metabolomic and proteomic analyses underscored the critical roles of photosynthetic and antioxidant pathways. Salt stress significantly decreased the net photosynthetic rate (Pn) by 67.7%, disrupted chloroplast ultrastructure, and reduced chlorophyll content by 41.6%. Conversely, MT treatment notably mitigated these detrimental effects. Moreover, MT enhanced the activities of antioxidant enzymes by approximately 10–20% and reduced the accumulation of oxidative stress markers by around 10–25% in maize seedlings under salt stress. In conclusion, this study conducted a systematic and multidimensional investigation into the mitigation of salt stress in maize seedlings by MT. Our results revealed that MT enhances antioxidant systems, increases chlorophyll content, and alleviates damage to chloroplast ultrastructure, thereby improving photosystem II performance and strengthening photosynthesis. This ultimately manifests as improved seedling phenotypes under salt stress. These findings provide a meaningful entry point for breeding salt-tolerant maize varieties and mitigating the adverse effects of salinized soil on maize growth and yield.

## 1. Introduction

Soil salinization poses a significant challenge to agricultural production [1], acting as a primary factor that threatens plant growth and development and restricts yield increases [2]. The average yield of major crops typically reaches only 20% to 50% of their historical maximums, largely due to the adverse effects of high soil salinity [3]. Salt stress disrupts plant growth via diverse mechanisms, including Na^+^ toxicity, nutrient imbalance, physiological drought, metabolic dysregulation, oxidative damage, and photoinhibition [4,5,6,7]. Plants have evolved intricate adaptive mechanisms to counteract salt stress by modulating transcriptional regulation, thereby influencing morphology, physiology, and metabolites [8].

Maize (*Zea mays* L.), the world’s third-largest staple crop, confronts abiotic stresses such as climate change and soil salinization [9]. Maize exhibits moderate salt tolerance, yet displays heightened sensitivity to salinity stress during seed germination and seedling stages compared to other growth phases. At a salinity concentration of 100 mM, maize seedling biomass decreases by approximately 50% [10]. Under abiotic stress, endogenous protective mechanisms in plants may be insufficient to counteract damage induced by salt stress [11]. Consequently, employing exogenous biostimulants or growth hormones to mitigate the impacts of abiotic stress on plants represents an effective novel strategy.

Melatonin (N-acetyl-5-methoxytryptamine, MT), a bioactive hormone, plays a pivotal role in the life processes of both animals and plants, encompassing circadian rhythm regulation, antioxidant capacity enhancement, and immune system strengthening [12,13]. Research has demonstrated that exogenous MT application can ameliorate photosynthesis in tomato (*Solanum lycopersicum*) plants under salt stress by elevating chlorophyll and carotenoid contents, as well as the maximum quantum yield of photosystem II photochemistry [14]. Specifically, a 70 µM MT treatment under salt stress conditions has been shown to increase chlorophyll levels and reduce reactive oxygen species production in barley (*Hordeum vulgare* L.), thereby mitigating oxidative damage and enhancing photosynthetic efficiency [15]. Furthermore, melatonin supplementation under salt stress can elevate potassium (K^+^) content, maintain cellular Na^+^/K^+^ homeostasis, and bolster plant salt tolerance [16]. However, current investigations into MT-mediated alleviation of salt stress predominantly focus on physiological aspects, with limited multi-omics correlation analysis, and the underlying mechanisms of MT in mitigating salt stress during the seedling stage of maize remain to be systematically elucidated.

Plants employ coordinated regulatory mechanisms at multiple physiological and molecular levels to respond to various abiotic stresses [17]. In previous studies, Su et al. found the combined application of exogenous melatonin and betaine could mitigate the adverse effects of NaCl stress on maize seed germination and seedling growth by regulating osmotic balance and enhancing the antioxidant system [18]. Ren et al. reported that exogenous melatonin improved salt tolerance in maize seedlings by alleviating osmotic, ionic, and oxidative stresses [19]. Zhang et al. showed that 100 µmol/L melatonin (MT) reduced the impact of salt stress on maize seed germination, with significantly improved germination and vigor indices compared to the salt-treated group [20]. Li et al. revealed that melatonin enhanced maize germination, growth, and salt tolerance by modulating reactive oxygen species accumulation and the antioxidant system [21]. Chen et al. demonstrated that exogenous MT improved salt tolerance in maize seedlings by enhancing antioxidant and photosynthetic capacities [22]. Wang et al., through transcriptomic and physiological experiments, indicated that melatonin pretreatment enhanced salt tolerance during maize seed germination by regulating antioxidant enzyme activities, endogenous hormone levels, and the expression of germination-related genes [23].

Previous studies on melatonin-alleviated salt stress in maize have predominantly focused on physiological or single-omics (transcriptomic) approaches. This study, through multi-omics integration combined with physiological experiments, partially validated prior findings. Given that proteomics, downstream of genomics and transcriptomics, serves as the direct executor of gene functions, it provides a more direct reflection of gene functional implementation compared to previous transcriptomic studies [24]. Metabolomics, positioned downstream of protein regulatory networks, offers a more direct representation of terminal biological activity information and is most closely associated with phenotypic changes, outperforming transcriptomic approaches in this regard [25]. Furthermore, multi-omics integration facilitates biomarker discovery and application: differentially expressed proteins or specific metabolites typically exhibit more direct functional associations with phenotypes compared to transcripts. Key differentially expressed proteins and metabolites identified through integrated analysis can serve as more reliable and sensitive molecular markers or diagnostic targets for evaluating melatonin-alleviated effects. Notably, the identified key functional proteins or their regulated metabolites are more suitable than gene sequences as direct targets for exogenous regulatory substance interventions.

## 2. Results

### 2.1. Melatonin Alleviates Salt-Induced Stress in Maize Seedlings

**Figure 1:**
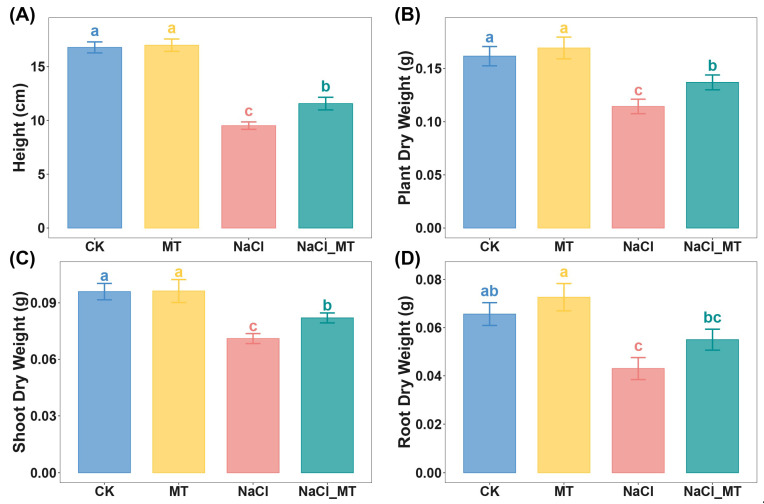
(**A**) Plant height, (**B**) plant dry weight, (**C**) shoot dry weight, and (**D**) root dry weight under normal cultivation, MT treatment, NaCl treatment, and NaCl_MT treatment conditions. Data are presented as mean ± standard deviation (sd). Each group had 3 biological replicates. Statistical methods employed were ANOVA followed by Tukey’s test or the Kruskal–Wallis H Test with Dunn’s Test, depending on the data distribution. Letters denote significant differences (a > b > c).

In this study, 1 µM MT had no significant impact on the height, plant dry weight, shoot dry weight, or root dry weight of maize seedlings compared to the control (CK) (Figure 1 and Figure S4). Conversely, 100 mM NaCl treatment markedly reduced these parameters. Cotreatment with 100 mM NaCl and 1 µM MT alleviated the salt-induced detrimental effects on these growth metrics. The underlying mechanisms of this alleviating effect warrant further investigation.

### 2.2. Identification of Key Metabolites and Metabolic Enrichment Analysis in Melatonin-Mediated Alleviation of Salt Stress

**Figure 2:**
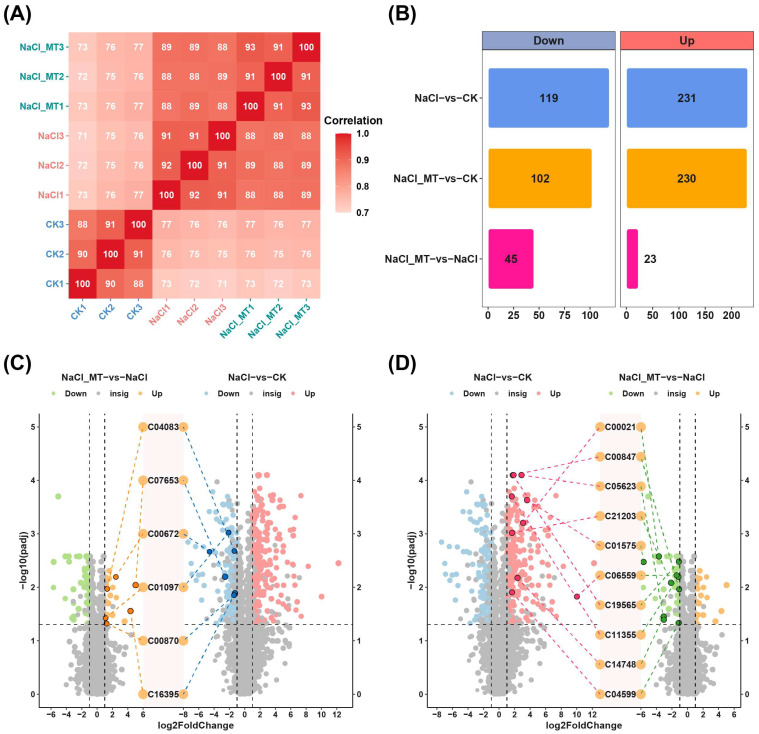
Overview of metabolomics data. (**A**) Correlation heatmap of metabolites across different groups. (**B**) Number of upregulated and downregulated differential metabolites in different comparison groups. (**C**) Differential metabolites that are downregulated in the NaCl treatment compared to normal cultivation but upregulated in the NaCl_MT treatment compared to the NaCl treatment. (**D**) Differential metabolites that are upregulated in the NaCl treatment compared to normal cultivation but downregulated in the NaCl_MT treatment compared to the NaCl treatment. The vertical gray dashed lines represent fold change = ±2, while the horizontal gray dashed line represents padj = 0.05.

To elucidate the key metabolites involved in MT-mediated alleviation of salt stress, metabolomic analysis was performed on the CK, NaCl, and NaCl_MT groups. Correlation analysis of metabolites across replicates within each group revealed high correlations and satisfactory sample reproducibility (Figure 2A). Differential metabolite analysis identified 199 downregulated (NaCl−vs−CK_Down) and 231 upregulated metabolites (NaCl−vs−CK_Up) in the NaCl group compared to CK (Figure 2B). In the NaCl_MT group, 102 metabolites were downregulated (NaCl_MT−vs−CK_Down) and 230 were upregulated (NaCl_MT−vs−CK_Up) compared to CK. Compared to NaCl, the NaCl_MT group exhibited 45 downregulated (NaCl_MT−vs−NaCl_Down) and 23 upregulated (NaCl_MT−vs−NaCl_Up) metabolites. The following metabolites exhibited downregulation in NaCl vs. CK but upregulation in NaCl_MT vs. NaCl: C04083 (N-(3-Methylbut-2-EN-1-YL)-9H-purin-6-amine), C07653 (Flutamide), C00672 (Deoxyribose 1-phosphate), C01097 (D-Tagatose 6-phosphate), C00870 (4-Nitrophenol), and C16395 (2-Amino-4,6-dinitrotoluene) (Figure 2C). Conversely, C00021 (S-adenosylhomocysteine, SAH), C00847 (Pyridoxate), C05623 (Isoquercitrin), C21203 (Geranyl Phosphate), C01575 (Ephedrine), C06559 (6-Chloro-N-(1-methylethyl)-1,3,5-triazine-2,4-diamine), C19565 (4-Hydroxy-1-(3-pyridinyl)-1-butanone), C11355 (4-amino-4-deoxychorismate), C14748 (20-Hydroxyeicosatetraenoic acid), and C04599 (1-Methyl-4-phenyl-1,2,3,6-tetrahydropyridine) showed upregulation in NaCl vs. CK but downregulation in NaCl_MT vs. NaCl (Figure 2D). These metabolites may serve as pivotal factors in melatonin-mediated alleviation of salt stress. Notably, among the aforementioned metabolites, only N-(3-Methylbut-2-EN-1-YL)-9H-purin-6-amine, Deoxyribose 1-phosphate, Pyridoxate, Isoquercitrin, Geranyl Phosphate, 4-Hydroxy-1-(3-pyridinyl)-1-butanone, and 1-Methyl-4-phenyl-1,2,3,6-tetrahydropyridine achieved MSI level 1 (Table S2).

**Figure 3:**
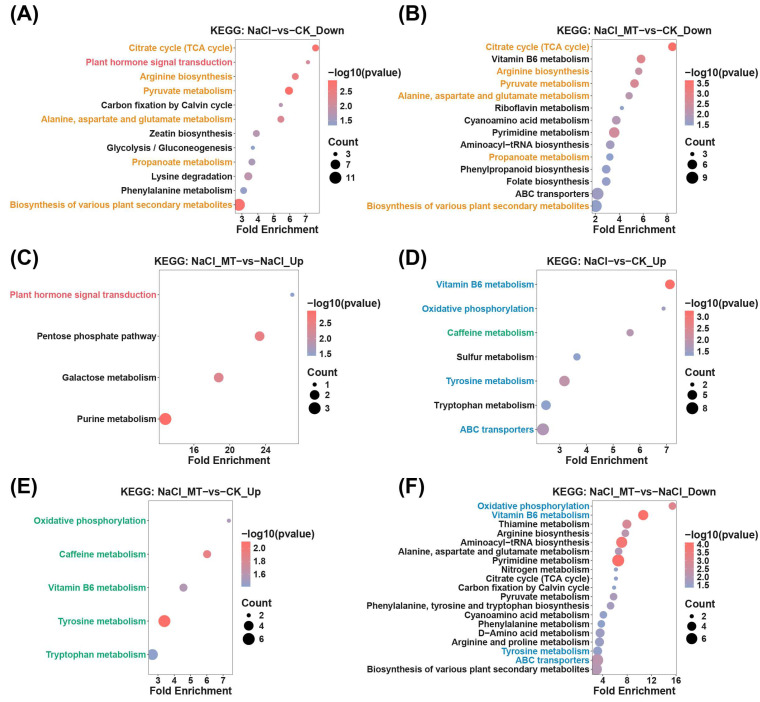
KEGG enrichment analysis of differential metabolites. (**A**) Enrichment analysis of downregulated differential metabolites in NaCl treatment compared to normal cultivation. (**B**) Enrichment analysis of downregulated differential metabolites in NaCl_MT treatment compared to normal cultivation. (**C**) Enrichment analysis of upregulated differential metabolites in NaCl_MT treatment compared to NaCl treatment. (**D**) Enrichment analysis of upregulated differential metabolites in NaCl treatment compared to normal cultivation. (**E**) Enrichment analysis of upregulated differential metabolites in NaCl_MT treatment compared to normal cultivation. (**F**) Enrichment analysis of downregulated differential metabolites in NaCl_MT treatment compared to NaCl treatment. Pathways annotated in orange are those shared between (**A**,**B**); pathways annotated in red are those shared between (**A**,**C**); pathways annotated in green are those shared between (**D**,**E**); pathways annotated in blue are those shared between (**D**,**F**).

Subsequently, KEGG enrichment analysis was performed on the metabolites. Pathways commonly enriched in both NaCl_MT−vs−NaCl and NaCl−vs−CK comparisons were identified as key pathways responding to salt stress yet insufficiently alleviated by MT. Conversely, pathways commonly enriched in NaCl_MT−vs−CK and NaCl−vs−CK comparisons represented critical pathways responding to salt stress and effectively mitigated by MT. Specifically, “Plant hormone signal transduction” was significantly enriched in both NaCl−vs−CK_Down and NaCl_MT−vs−NaCl_Up comparisons (Figure 3A,C). “Citrate cycle (TCA cycle)”, “Arginine biosynthesis”, “Pyruvate metabolism”, “Alanine, aspartate and glutamate metabolism”, “Propanoate metabolism”, and “Biosynthesis of various plant secondary metabolites” were notably enriched in both NaCl_MT−vs−CK_Down and NaCl_MT−vs−NaCl_Up comparisons (Figure 3A,B). “Oxidative phosphorylation”, “Caffeine metabolism”, “Vitamin B6 metabolism”, “Tyrosine metabolism”, and “Tryptophan metabolism” exhibited significant enrichment in both NaCl_MT−vs−CK_Up and NaCl−vs−CK_Up comparisons (Figure 3D,E). Additionally, “Oxidative phosphorylation”, “Vitamin B6 metabolism”, “Tyrosine metabolism”, and “ABC transporters” were commonly enriched in NaCl_MT−vs−NaCl_Down and NaCl−vs−CK_Up comparisons (Figure 3D,F).

### 2.3. Proteomic Analysis and Protein-Level Enrichment Analysis

To further elucidate the underlying mechanisms by which MT alleviates salt stress at the protein level, we conducted proteomic sequencing on CK, NaCl, and NaCl_MT groups. Principal component analysis (PCA) of the normalized abundance of proteins across groups revealed distinct clustering in different quadrants (Figure S5A). Concurrently, hierarchical clustering heatmaps demonstrated that within-group replicates clustered together, with the NaCl group being the most distant from the CK group and the NaCl_MT group positioned intermediate between NaCl and CK (Figure S5B). This indicates that salt stress induces significant protein-level changes compared to CK, which are partially mitigated by MT treatment.

**Figure 4:**
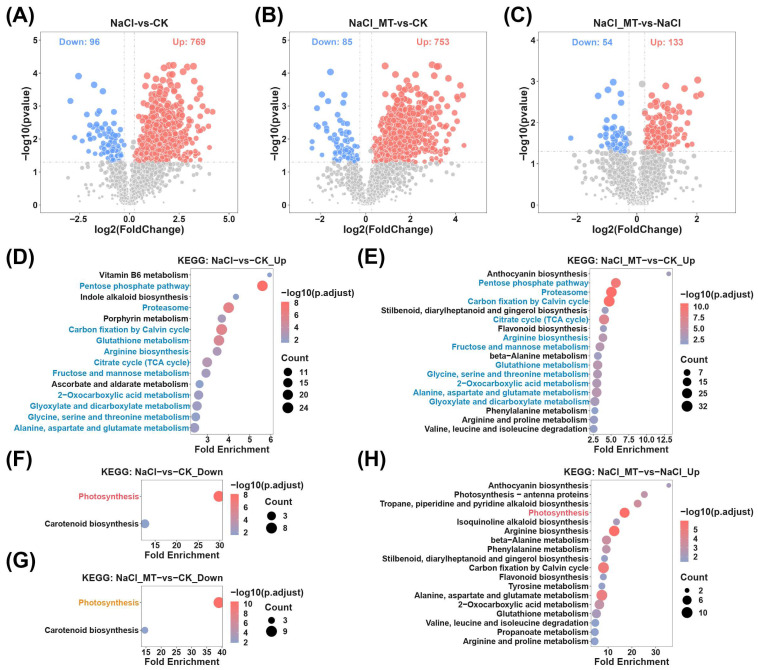
(**A**–**C**) Volcano plots of differential proteins in each comparison group. (**D**–**H**) KEGG enrichment analysis of differential proteins. Red, blue, and grey circles are used to represent up-regulated, down-regulated, and not significant differential proteins, respectively. The vertical gray dashed lines represent fold change = ±1.2, while the horizontal gray dashed line represents padj = 0.05. (**D**) Enrichment analysis of upregulated differential proteins in NaCl treatment compared to normal cultivation. (**E**) Enrichment analysis of upregulated differential proteins in NaCl_MT treatment compared to normal cultivation. (**F**) Enrichment analysis of downregulated differential proteins in NaCl treatment compared to normal cultivation. (**G**) Enrichment analysis of downregulated differential proteins in NaCl_MT treatment compared to normal cultivation. (**H**) Enrichment analysis of upregulated differential proteins in NaCl_MT treatment compared to NaCl treatment. Pathways annotated in blue are those shared between (**D**,**E**); pathways annotated in orange are those shared between (**F**,**G**); pathways annotated in red are those shared between (**F**,**H**).

Differential protein analysis revealed that NaCl-vs-CK exhibited 96 downregulated and 769 upregulated differentially expressed proteins (DEPs) (Figure 4A), while NaCl_MT-vs-CK displayed 85 downregulated and 753 upregulated DEPs (Figure 4B). Comparing NaCl_MT-vs-NaCl, 54 DEPs were downregulated and 133 were upregulated (Figure 4C). KEGG enrichment analysis of these DEPs identified shared significant enrichment in pathways including “Pentose phosphate pathway”, “Proteasome”, “Carbon fixation by Calvin cycle”, “Glutathione metabolism”, “Arginine biosynthesis”, “Citrate cycle (TCA cycle)”, “Fructose and mannose metabolism”, “2-Oxocarboxylic acid metabolism”, “Glyoxylate and dicarboxylate metabolism”, “Glycine, serine and threonine metabolism”, and “Alanine, aspartate and glutamate metabolism” in both NaCl-vs-CK_Up and NaCl_MT-vs-CK_Up comparisons (Figure 4D,E). “Photosynthesis” was commonly and significantly enriched in NaCl-vs-CK_Down, NaCl_MT-vs-CK_Down, and NaCl_MT-vs-NaCl_Up (Figure 4F–H), indicating photosynthetic impairment under salt stress, which persisted in NaCl_MT treatment relative to CK but was alleviated compared to NaCl stress. Notably, no significantly enriched KEGG pathways were identified in the NaCl_MT-vs-NaCl_Down comparison group.

### 2.4. Integrated Metabolomic and Proteomic Analysis Reveals Key Mechanisms Underlying Melatonin’s Mitigating Effects

Integrating the results of proteomic and metabolomic enrichment analyses, we categorized the pathways into two groups: those responsive to salt stress and significantly alleviated by MT, and those responsive to salt stress but not significantly alleviated by MT. These pathways are involved in multiple biological processes, including signal transduction, substance and energy metabolism, photosynthesis, and antioxidant defense. The pathways responsive to salt stress and significantly alleviated by MT comprise pathways including “Plant hormone signal transduction”, “Oxidative phosphorylation”, “Vitamin B6 metabolism”, “Tyrosine metabolism”, “ABC transporters”, and “Photosynthesis”. In contrast, the pathways responsive to salt stress yet not significantly alleviated by MT encompass pathways such as “Citrate cycle (TCA cycle)”, “Arginine biosynthesis”, “Pyruvate metabolism”, “Alanine, aspartate and glutamate metabolism”, “Propanoate metabolism”, “Biosynthesis of various plant secondary metabolites”, “Caffeine metabolism”, “Pentose phosphate pathway”, “Proteasome”, “Carbon fixation by Calvin cycle”, “Glutathione metabolism”, “Fructose and mannose metabolism”, “2−Oxocarboxylic acid metabolism”, “Glyoxylate and dicarboxylate metabolism”, and “Glycine, serine and threonine metabolism”.

**Figure 5:**
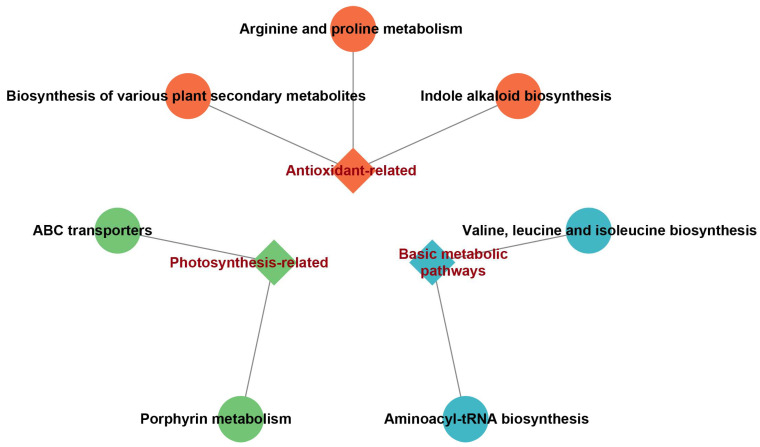
Enrichment analysis results of the highest-scoring subnetwork within the interaction network of differential proteins and differential metabolites.

Subsequently, we performed Pearson correlation analysis between differentially expressed proteins (DEPs) and metabolites (DEMs), followed by construction of a correlation network for protein-metabolite pairs meeting the selection criteria (Figure S6A). The MCODE algorithm identified the highest-scoring subnetwork comprising 48 nodes and 460 edges (Figure S6B). KEGG pathway enrichment analysis of the subnetwork constituents revealed seven significantly enriched pathways, categorized into three functional groups: (1) photosynthesis-related pathways (ABC transporters; porphyrin metabolism); (2) antioxidant-related pathways (arginine and proline metabolism; biosynthesis of plant secondary metabolites; indole alkaloid biosynthesis); and (3) basic metabolic pathways (valine, leucine, and isoleucine biosynthesis; aminoacyl-tRNA biosynthesis) (Figure 5). This result indicates that the proteins and metabolites within the subnetwork showing the strongest correlation collectively function in photosynthesis-related and antioxidant-related pathways.

### 2.5. Melatonin Alleviates Drought-like Salt Stress Effects in Maize Seedlings by Enhancing Photosynthesis

**Figure 6:**
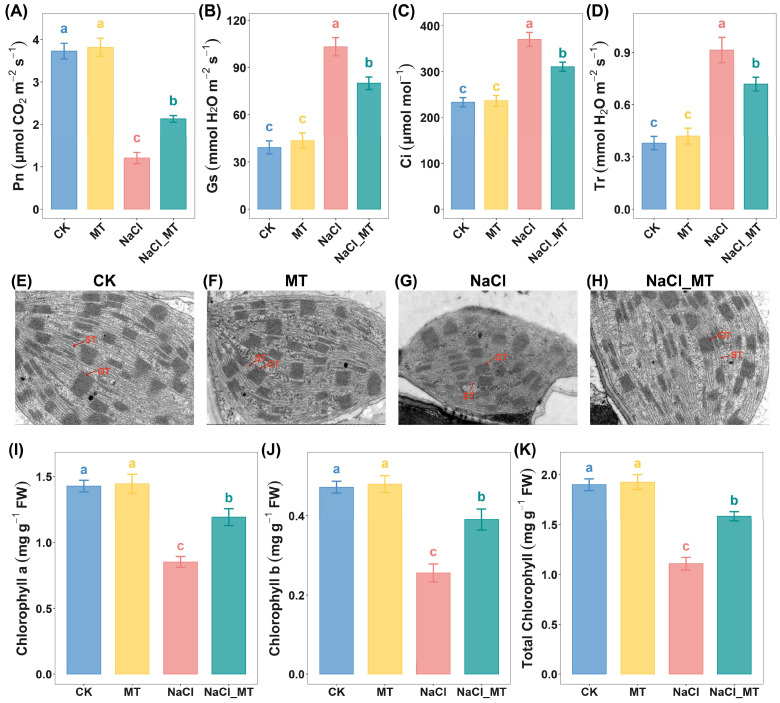
Melatonin alleviated the negative effects of salt stress on photosynthesis. (**A**–**D**) Photosynthetic parameters under normal cultivation, MT treatment, NaCl treatment, and NaCl_MT treatment: (**A**) Net photosynthetic rate, (**B**) Stomatal conductance, (**C**) Intercellular carbon dioxide concentration, (**D**) Transpiration rate. (**E**–**H**) Ultrastructural images of chloroplasts at 1-micrometer magnification under different treatment groups. GT, grana thylakoid; ST, stroma thylakoid. (**I**–**K**) Chlorophyll contents under normal cultivation, MT treatment, NaCl treatment, and NaCl_MT treatment: (**I**) Chlorophyll a, (**J**) Chlorophyll b, (**K**) Total chlorophyll. Data are presented as mean ± standard deviation (sd). Each group had 5 biological replicates for photosynthetic parameters and 3 for chlorophyll contents. Statistical methods employed were ANOVA followed by Tukey’s test or the Kruskal–Wallis H Test with Dunn’s Test, depending on the data distribution. Letters denote significant differences (a > b > c).

Among the pathways identified in our joint analysis, two were associated with photosynthesis. To validate the critical role of photosynthesis in melatonin-mediated alleviation of salt stress in maize seedlings, we measured key photosynthetic parameters. NaCl treatment significantly reduced net photosynthetic rate (Pn) (Figure 6A), water-use efficiency (WUE), and instantaneous water-use efficiency (iWUE) (Figure S7) compared to CK, while MT significantly mitigated this decline. Additionally, NaCl treatment increased stomatal conductance (Gs) (Figure 6B), intercellular CO_2_ concentration (Ci) (Figure 6C), and transpiration rate (Tr) (Figure 6D) compared to CK, all of which were significantly restored by MT. Notably, none of these parameters differed significantly between the MT-treated and CK groups.

Transmission electron microscope (TEM) revealed that chloroplasts in CK and MT groups exhibited large, regular ellipsoidal shapes, whereas NaCl-stressed chloroplasts were smaller and more fusiform (Figure S8 and Figure 6E–H). MT supplementation partially reversed NaCl-induced chloroplast morphological changes. Ultrastructurally, CK and MT chloroplasts displayed abundant, well-stacked grana thylakoids with ordered lamellae and continuous stroma thylakoids, forming a continuous membrane network connecting grana. In contrast, NaCl treatment reduced grana thylakoid abundance, disrupted stacking, and caused stroma thylakoid fragmentation or swelling. NaCl_MT treatment partially restored grana and stroma thylakoid organization, with improved stacking and reduced swelling.

Chlorophyll content analysis further confirmed that NaCl treatment significantly decreased chlorophyll a, chlorophyll b, and total chlorophyll levels compared to CK, whereas MT supplementation significantly attenuated these reductions (Figure 6I–K and Figure S9). Table 1 presents 10 biophysical parameters of photosystem II (PSII) behavior derived from the JIP-test, elucidating the disruption of PSII in leaves under salt stress and the mitigating effect of MT. Salt stress significantly decreased the Fv/Fm, Fv/Fo, ψEo, and φEo values compared to CK, while Vj, Vi, ABS/RC, DIo/RC, and TRo/RC values were markedly elevated. Notably, in the NaCl_MT treatment group, MT effectively mitigated the effects induced by NaCl alone, driving these parameters closer to the CK levels. The Fv/Fm ratio is commonly used to assess the maximum photochemical efficiency of PSII. When the Fv/Fm ratio falls below 0.8, it indicates that the plant is under stress, with severely inhibited growth and development [26]. The Fv/F0 ratio represents PS II activity and is typically employed to evaluate photosynthetic efficiency under varying environmental conditions [27]. These two ratios provide information from different perspectives, and a decline in both Fv/Fm and Fv/F0 ratios under stress conditions generally suggests structural and functional alterations in photosynthetic components.

### 2.6. Melatonin Enhances Antioxidant Defense in Seedlings to Promote Salt Stress Tolerance

**Figure 7:**
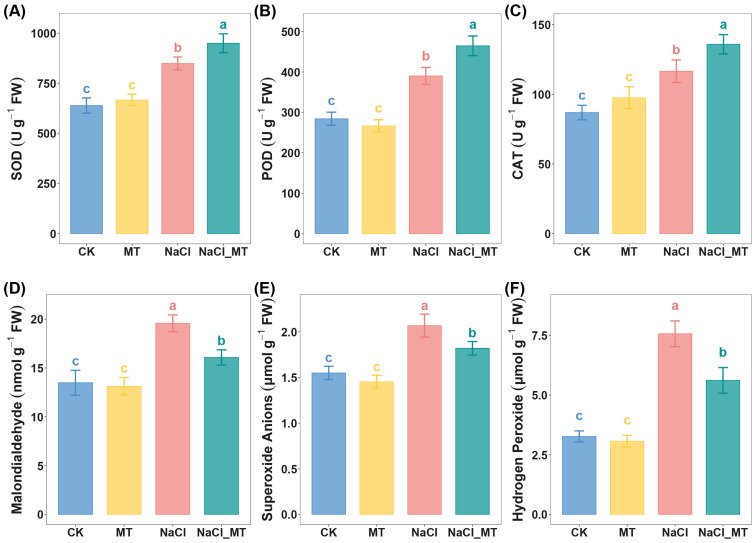
Melatonin alleviated oxidative stress under salt stress. (**A**–**C**) Activities of antioxidant enzymes under normal cultivation, MT treatment, NaCl treatment, and NaCl_MT treatment: (**A**) Superoxide dismutase activity, (**B**) Peroxidase activity, (**C**) Catalase activity. (**D**–**F**) Contents of oxidative stress substances under normal cultivation, MT treatment, NaCl treatment, and NaCl_MT treatment: (**D**) Malondialdehyde, (**E**) Superoxide Anions, (**F**) Hydrogen Peroxide. Data are presented as mean ± standard deviation (sd). Each group had 3 biological replicates. Statistical methods employed were ANOVA followed by Tukey’s test or the Kruskal–Wallis H Test with Dunn’s Test, depending on the data distribution. Letters denote significant differences (a > b > c).

In addition to the integrated metabolomic and proteomic analyses revealing three antioxidant-related pathways, we verified the pivotal role of antioxidants in melatonin-mediated alleviation of salt stress by assessing antioxidant enzyme activities and oxidative stress marker levels. Under salt stress (NaCl), activities of superoxide dismutase (SOD), peroxidase (POD), and catalase (CAT) were significantly elevated compared to CK, with further enhancement observed upon NaCl_MT treatment (Figure 7A–C). DAB staining intensity (correlating with H_2_O_2_ accumulation) and NBT staining (indicating O_2_·^−^ levels) were darker in NaCl-treated seedlings compared to CK and MT alone, while NaCl_MT treatment reduced staining intensity relative to NaCl alone (Figure S10A,B). Quantitative measurements confirmed that NaCl increased MDA, O_2_·^−^, and H_2_O_2_ contents compared to CK, and these increases were significantly mitigated by NaCl_MT co-treatment (Figure 7D–F).

## 3. Discussion

Soil salinization exerts profound impacts on crop yields. Maize, one of the world’s staple food crops, exhibits heightened sensitivity to salinity stress during its seedling stage. Although MT has been previously demonstrated to alleviate salt stress in plants, its underlying mechanisms in enhancing salt stress tolerance in maize seedlings remain incompletely elucidated.

In this study, metabolomic analysis identified several important metabolites potentially pivotal in melatonin-mediated alleviation of salt stress. D-Tagatose 6-phosphate, participating in glycolysis, the pentose phosphate pathway, or fructose metabolism, may serve as a precursor for compatible solute (e.g., proline, betaine) synthesis, thereby regulating cellular osmotic pressure [28]. Geranyl Phosphate, a precursor for terpenoid synthesis involved in gibberellin and carotenoid biosynthesis [29], was upregulated under salt stress to promote protective terpenoid synthesis but downregulated upon melatonin-induced stress alleviation, possibly due to reduced terpenoid demand. Isoquercitrin, a flavonoid antioxidant, scavenges ROS under salt stress [30], while melatonin, acting as a direct ROS scavenger, synergistically upregulates other antioxidant enzymes, reducing reliance on isoquercitrin. S-Adenosylhomocysteine (SAH), a methyl cycle metabolite, inhibits RNA methylation [31]. High salinity suppresses the methyl cycle, leading to SAH accumulation, which blocks methyltransferase activity and affects epigenetic regulation [32]. Melatonin may promote homocysteine remethylation, thereby reducing SAH levels, maintaining epigenetic homeostasis. The mechanisms underlying other metabolites warrant further investigation.

Subsequently, correlation analysis between differentially expressed proteins and metabolites was performed to extract the largest subnetwork from the correlation network for enrichment analysis. The enrichment results were categorized into three major groups: photosynthesis-related, antioxidant-related, and basic metabolic pathways. Most ABC transporters are membrane-bound proteins distributed across multiple subcellular structures, including the cell membrane, chloroplasts, mitochondria, vacuoles, and peroxisomes [33]. Utilizing the energy released from ATP hydrolysis, they mediate the transmembrane transport of various substances, such as hormones, secondary metabolites, and heavy metal ions, thereby regulating numerous critical physiological processes in organisms [34,35,36]. For instance, within the photosynthesis-related pathways, ABC transporters play pivotal roles in regulating metal ion homeostasis, participating in the synthesis and transport of photosynthetic pigments, and maintaining chloroplast membrane integrity [37]. Porphyrin metabolism, a core pathway for chlorophyll biosynthesis, yields magnesium porphyrin, a critical cofactor in the light reactions of photosynthesis [38]. Melatonin may influence the porphyrin metabolism pathway to sustain normal chlorophyll content and chloroplast structure, thereby ensuring efficient photosynthesis in maize under salt stress [39]. In terms of antioxidant defense, the enrichment analysis highlighted three pathways: arginine and proline metabolism; biosynthesis of plant secondary metabolites; and indole alkaloid biosynthesis. Proline, a key compatible solute in plants, accumulates under osmotic stress to maintain cell turgor, stabilize membrane structures, and directly scavenge hydroxyl radicals [40]. Additionally, proline can enhance ROS scavenging capacity by inducing the expression of antioxidant enzymes [41]. In the biosynthesis of plant secondary metabolites, salt stress often triggers the synthesis of flavonoids, terpenoids, and other secondary metabolites to counteract oxidative stress [42]. The synthesis of these metabolites requires substantial amounts of NADPH, which melatonin may provide by regulating the pentose phosphate pathway (PPP). These secondary metabolites can directly scavenge ROS or reduce oxidative damage by chelating metal ions to mitigate Fenton reactions [43]. Indole alkaloids, with their endogenous antioxidant activities, can be synthesized more efficiently in plants upon melatonin treatment [44], thereby enhancing the plant’s antioxidant capacity.

Salt stress, a prevalent environmental issue, disrupts the redox balance and metabolic processes in plants, leading to excessive accumulation of ROS, such as O_2_·^−^ and H_2_O_2_ [45]. This overaccumulation triggers membrane lipid peroxidation, compromising the integrity of cellular membranes [46]. As the primary product of membrane lipid peroxidation, MDA content directly reflects the extent of oxidative damage to cells [47]. Exogenous MT application mitigates the adverse effects of NaCl stress by enhancing antioxidant enzyme activities in maize seedling leaves. Specifically, MT significantly increases the activities of SOD, POD, and CAT under NaCl stress. SOD converts O_2_·^−^ into H_2_O_2_, which is subsequently decomposed into H_2_O and O_2_ by POD and CAT, effectively reducing ROS concentration [48,49]. This cascade of enzymatic reactions lowers ROS accumulation, thereby inhibiting lipid peroxidation chain reactions and alleviating ROS-induced membrane damage, as evidenced by a significant decrease in MDA content.

In this study, under NaCl stress alone, the Pn of seedlings significantly decreased compared to the control group, whereas NaCl_MT treatment significantly increased Pn relative to NaCl stress alone. Interestingly, NaCl stress significantly elevated Gs, intercellular Ci, and Tr compared to the control group, while NaCl_MT treatment attenuated these increases. Similar phenomena were observed in the salt-tolerant cultivar Zhengdan 958, which initially or under moderate NaCl stress maintains short-term water and gas exchange by actively opening stomata; however, with prolonged or intensified stress, Gs and Tr gradually decline [50]. Increased Gs leads to elevated Ci and Tr, but despite the ample CO_2_ supply indicated by higher Ci, Pn may decrease significantly due to photosystem damage.

Thylakoid membranes in higher plant chloroplasts consist of two distinct structural domains: stacked grana and stroma lamellae, housing the complete photosynthetic electron transport chain, including photosystem I (PSI) reaction centers in stroma thylakoids and photosystem II (PSII) reaction centers in granal thylakoids [51,52,53]. To verify whether the decline in Pn under NaCl stress resulted from non-stomatal limitations due to photosystem damage, we first conducted TEM analysis on seedling leaves. TEM revealed partial disintegration of thylakoid structures under NaCl stress, with disordered granal thylakoid arrangement and swollen or ruptured stroma lamellae; these detrimental effects were mitigated by NaCl_MT treatment. In higher plants, PSII is a multi-subunit pigment-protein complex located in chloroplast thylakoid membranes [54]. Granal thylakoids serve as the core sites for PSII light harvesting, water splitting, and proton release, while stroma lamellae facilitate electron transport chain extension, PSII repair, and light energy distribution. Alterations in chloroplast ultrastructure are partly attributed to excessive ROS accumulation, which damages thylakoids via lipid peroxidation, leading to membrane leakage and loss of integrity [55,56]. Additionally, ROS accumulation reduces chlorophyll content [57]. Measurements showed significant declines in chlorophyll a, chlorophyll b, and total chlorophyll under NaCl stress, with NaCl_MT treatment attenuating these reductions. Chlorophyll a and chlorophyll b play complementary and critical roles in PSII: chlorophyll a, as the core pigment, directly participates in photochemical reactions, while chlorophyll b, as a light-harvesting pigment, expands spectral absorption range and optimizes light energy utilization. Their coordinated energy transfer and dynamic regulation ensure efficient PSII function and smooth photosynthesis. Thus, structural changes in thylakoids and reduced chlorophyll content likely impair PSII function, suppressing photosynthesis and manifesting as decreased Pn [58]. This was further confirmed by chlorophyll a fluorescence kinetics: the maximum photochemical efficiency (Fv/Fm), a key indicator of PSII potential efficiency, significantly declined under NaCl stress compared to the control group but increased with NaCl + MT treatment.

In summary, this study conducted a systematic and multidimensional investigation into how exogenous melatonin alleviates salt stress in maize seedlings by integrating physiology, metabolomics, and proteomics, offering a more comprehensive and systematic biological perspective for this research field. Our findings partially corroborated previous studies mainly based on physiology and transcriptomics. Given that proteomics and metabolomics are positioned downstream in the central dogma, they better reflect gene functional execution and terminal information of life activities, closely relating to changes in biological phenotypes. Based on the significantly enriched pathways (photosynthesis-related pathways and antioxidant-related pathways) identified through multi-omics analysis, physiological experiments were carried out. The results indicated that exogenous melatonin primarily enhances antioxidant systems, increases chlorophyll content, and mitigates damage to chloroplast ultrastructure, thereby improving PS II performance and subsequently strengthening photosynthesis. Collectively, this study demonstrates that exogenous melatonin can enhance salt tolerance in maize seedlings, ultimately manifesting as improved seedling phenotypes under salt stress.

## 4. Materials and Methods

### 4.1. Plant Materials, Chemicals, and Experimental Design

The seeds of the high-yielding hybrid maize variety Zhengdan 958 were purchased from Yongfeng Agrochemical Co., Ltd (Kaifeng, China). Prior to germination, the seed coatings were removed by rinsing the seeds several times with distilled water. Subsequently, the seeds were surface-sterilized by immersing them in 0.1% (*w*/*v*) sodium hypochlorite (NaClO) solution for 10 min, followed by three rinses with distilled water to eliminate any residual NaClO. The sterilized seeds were then evenly spread in a seedling tray, moistened with an appropriate amount of distilled water, and incubated in the dark at 25 °C for germination. Two days later, germinated seeds with radicles emerging upward were transferred to a fresh seedling tray, kept moist with distilled water, and cultivated in a plant growth chamber under a 12-h light/12-h dark cycle at 25 °C (day) and 22 °C (night), with a light intensity of 300 µmol m^−2^ s^−1^ and relative humidity of 80%. After three days, uniformly growing seedlings were selected and transferred to an intelligent artificial climate chamber, where they were further cultivated under a 16-h light/8-h dark cycle at 28 °C (day) and 25 °C (night), maintaining the same light intensity and relative humidity for subsequent experiments.

After identifying the optimal concentrations of NaCl and melatonin (MT) (Text S1, Figures S1 and S2), four distinct treatment groups were established based on a half-strength Hoagland nutrient solution with varying additives: (1) Control group (CK): No NaCl or MT added; (2) MT treatment group (MT): Supplemented with 1 µM MT; (3) NaCl treatment group (NaCl): Supplemented with 100 mM NaCl; (4) NaCl + MT treatment group (NaCl_MT): Supplemented with both 100 mM NaCl and 1 µM MT. Notably, the optimal concentration identified in this study matched that reported in prior research [19,59]. To maintain a consistent nutrient volume, fresh half-strength Hoagland solution was added daily, and the entire solution was replaced with fresh solution of the same composition every two days. After four days of treatment, phenotypic data were collected, and the second leaves were harvested for subsequent experiments. For plant height measurement, a ruler was used to measure the height of the plant with leaves straightened. For dry weight measurement of different seedling parts, the processed seedlings were dried in a vacuum oven (DZG-6050SA, Senxin Experimental Instrument Co., Ltd., Shanghai, China) at 135 °C for 30 min, followed by drying at 75 °C for 12 h.

### 4.2. Metabolomic Analysis

Three biological replicates of leaves from three groups (CK, NaCl, and NaCl_MT) were analyzed. Samples were homogenized in methanol containing 4 ppm 2-Amino-3-(2-chloro-phenyl)-propionic acid (internal standard) using steel ball grinding (55 Hz, 60 s), followed by ultrasonication (15 min) and centrifugation (12,000 rpm, 4 °C, 10 min). Supernatants were filtered (0.22 µm) for LC-MS analysis. Separation was achieved on a Vanquish UHPLC system (Thermo Fisher, Waltham, MA, USA) with an ACQUITY HSS T3 column (2.1 × 100 mm, 1.8 µm) at 0.3 mL/min (Milford, MA, USA). Mobile phases: ESI(+)-0.1% FA in water/acetonitrile; ESI(-)-5 mM ammonium formate/acetonitrile. Both modes used identical 8-minute gradients: 10–98% organic phase (1–5 min), maintained (5–6.5 min), and re-equilibrated to 10% (6.6–8 min). Full scan (*m*/*z* 100–1000) and ddMS2 acquisitions were performed on Orbitrap Exploris 120 (60,000/15,000 resolution) with ESI± (±3.5/2.5 kV) (Waltham, MA, USA). Raw data were converted via ProteoWizard and analyzed using XCMS (centWave algorithm, 15 ppm tolerance). Metabolites were identified through HMDB, MassBank, KEGG, LipidMaps, and mzCloud matching, combining exact mass (<5 ppm) and MS/MS spectral alignment.

### 4.3. Label-Free Proteomic Analysis

Three biological replicates of leaves from three groups (CK, NaCl, and NaCl_MT) were analyzed. Protein samples were processed using label-free quantitative proteomics methodology. Briefly, samples were extracted using Tris/PVPP/glycerol buffer, followed by ultrafiltration concentration and BCA quantification. After reduction (10 mM DTT, 56 °C) and alkylation (50 mM IAA, dark), tryptic digestion (37 °C overnight) was performed. Peptides were desalted using C18 columns and analyzed via Q Exactive HF-X Orbitrap coupled to Easy-nLC 1200. Chromatographic separation used a 15 cm C18 column (1.9 µm) with a 90-minute gradient (4–40% ACN/0.1% FA) at 600 nL/min. MS acquisition included full scans (70,000 resolution, *m*/*z* 300–1800) and top20 HCD-MS/MS (28% NCE). Data were processed through MaxQuant (v1.6.2.10) with trypsin specificity (2 missed cleavages), 20 ppm mass tolerance, carbamidomethylation (fixed), and oxidation/N-terminal acetylation (variable). Proteins were identified at 1% FDR with ≥1 unique peptide.

### 4.4. Identification and Analysis of Metabolites and Proteins

We retained proteins or metabolites with values greater than 0 in at least three groups and performed normalization. For metabolomics, we employed the PLS-DA model to calculate VIP scores, with the model exhibiting an R^2^X of 0.641, an R^2^Y of 0.998, and a Q^2^ of 0.929. The criteria for selecting differential metabolites were set as a Benjamini–Hochberg (BH)-adjusted *p*-value < 0.05, an absolute fold change (FC) > 2, and a VIP score > 1. For differential proteins, the criteria included a BH-adjusted *p*-value < 0.05 and an absolute FC > 1.2. Enrichment analysis was performed using the clusterProfiler package (version 4.14.6) [60], with pathways deemed significantly enriched if they exhibited a BH-adjusted *p*-value < 0.05. Pearson correlation analysis was conducted between differential metabolites and proteins, followed by the construction of a correlation network using Cytoscape (version 3.10.1) [61]. Correlations were filtered based on a BH-adjusted *p*-value < 0.001 and an absolute correlation coefficient > 0.9. The most significant subnetwork was extracted using the MCODE plugin (version 2.0.3).

### 4.5. Chlorophyll Content Determination

Chlorophyll content was measured using a modified protocol based on previously described methods [62]. Fresh leaf tissue (0.1 g) from seedlings was finely chopped and immersed in 10 mL of extraction solvent (acetone:ethanol:water = 4.5:4.5:1) under dark conditions for 12–16 h, with intermittent vortexing (3–4 times) to ensure complete pigment dissolution. Absorbance was measured at 470 nm, 645 nm, and 663 nm using a spectrophotometer for calculation of the content of chlorophyll a (Ca), chlorophyll b (Cb), and total chlorophyll.

### 4.6. Measurement of Photosynthetic Parameters and Chlorophyll a Fluorescence Parameters

Using the CIRAS-4 portable photosynthesis measurement system (CIRAS-4, PP Systems, http://ppsystems.com/, Amesbury, MA, USA), five uniformly growing seedlings from each treatment group were selected to measure the following parameters on the second leaf: net photosynthetic rate (Pn), stomatal conductance (Gs), intercellular CO_2_ concentration (Ci), and transpiration rate (Tr). Following the method of Leakey et al., water-use efficiency (WUE) and instantaneous water-use efficiency (iWUE) were calculated as the ratios of Pn to Tr and Pn to Gs, respectively [63].

Chlorophyll a fluorescence was assessed using a Handy Plant Efficiency Analyzer (Handy PEA, Hansatech, Norfolk, UK), following the protocol outlined by previous research [64]. For each experimental treatment, the second fully expanded leaf from the apex was selected, with ten replicates per treatment. Prior to fluorescence measurements, these leaves were subjected to a 30-minute dark adaptation period. The key fluorescence parameters measured included: Fo, representing the minimal fluorescence yield; Fm, the maximal fluorescence yield; Fv, the variable fluorescence calculated as Fm−Fo; Fv/Fm, which quantifies the maximum photochemical efficiency of photosystem II (PS II); Fv/Fo, an indicator of the efficiency of electron transfer to the active reaction centers of PS II; Vj, the relative variable fluorescence at the J-step of the fluorescence induction curve; Vi, the relative variable fluorescence at the I-step; ABS/RC, the absorbed photon flux per active reaction center; DIo/RC, the energy dissipated per active reaction center; TRo/RC, the trapped energy flux per active reaction center; ETo/RC, the electron transport flux per active reaction center; φEo, the quantum yield of electron transport; and ΨEo, the probability that a trapped exciton can move an electron beyond the primary quinone acceptor into the electron transport chain. The details for these parameters can be found in Figure S3 and Table S1.

### 4.7. Chloroplast Ultrastructural Analysis

Fresh leaf tissues (approximately 1 mm^2^) of maize seedlings were fixed in 1% (*v*/*v*) glutaraldehyde at 4 °C for 3 days. Subsequently, the samples were fixed in 1% (*v*/*v*) osmium tetroxide solution at 4 °C for 2 h and dehydrated through a graded series of acetone solutions (30–90%). Following dehydration, the samples were progressively embedded in Epon-Araldite and polymerized in epoxy resin. Ultrathin sections (approximately 60–80 nm) were prepared, mounted on 300-mesh copper grids with formvar films, and stained sequentially with uranyl acetate for 30 min and lead citrate for 10 min. The ultrastructure of the samples was observed using a Hitachi 600A-2 transmission electron microscope (TEM) (Hitachi High-Tech Co., Tokyo, Japan).

### 4.8. DAB Staining for H_2_O_2_ Detection

The distribution and relative abundance of hydrogen peroxide (H_2_O_2_) in plant tissues were visualized using 3,3′-diaminobenzidine (DAB) staining (Solarbio, Beijing, China), which forms a reddish-brown precipitate upon reaction with H_2_O_2_. Fresh leaf tips were excised and fully immersed in pre-prepared DAB solution (1 mg/mL, pH 3.8) for 8 h in the dark at 25 °C. After incubation, leaves were boiled in 95% ethanol for 10 min to decolorize chlorophyll, rinsed thoroughly with distilled water, and photographed for analysis.

### 4.9. NBT Staining for O_2_·^−^ Detection

The distribution and relative abundance of superoxide anions (O_2_·^−^) in plant tissues were visualized using nitroblue tetrazolium (NBT) staining (Solarbio, Beijing, China), which produces a dark-blue formazan precipitate upon reaction with O_2_·^−^. Fresh leaf tips were excised and fully immersed in NBT solution (6 mM, pH 6.0) prepared in 10 mM sodium citrate buffer. Samples were incubated for 8 h at 25 °C under illumination, followed by boiling in 95% ethanol for 10 min to decolorize chlorophyll. After rinsing with distilled water, stained leaves were photographed for analysis.

### 4.10. SOD Activity Assay

Superoxide dismutase (SOD) activity was determined using the NBT method according to the manufacturer’s protocol (SOD Kit, Nanjing JC Biotech Co., Ltd., Nanjing, China). Briefly, samples were mixed with extraction buffer and sonicated, followed by centrifugation at 8000× *g* at 4 °C for 10 min to collect the supernatant. The reaction mixture (total volume: 200 µL) comprised 45 µL of Reagent 1, 2 µL of diluted Reagent 2, 18 µL of sample, 35 µL of Reagent 3, and 100 µL of diluted Reagent 4. After incubation at room temperature for 30 min, absorbance was measured at 560 nm using a spectrophotometer.

### 4.11. POD Activity Assay

Peroxidase (POD) activity was measured using a microplate method at 470 nm, as per the manufacturer’s instructions (Peroxidase Kit, Nanjing JC Biotech Co., Ltd.). Samples were preprocessed by sonication and centrifugation at 4 °C to obtain supernatants. Reagents 1, 2, and 3 were mixed according to the kit protocol, pre-warmed, and combined with the sample. Absorbance was recorded at 470 nm at 1 min (A_1_) and 2 min (A_2_), and ΔA = A_2_ − A_1_ was calculated.

### 4.12. CAT Activity Assay

Catalase (CAT) activity was measured using the ammonium molybdate microspectrophotometric method with a commercial kit (Solarbio Technology Co., Ltd., Beijing, China). Tissue samples were homogenized in extraction buffer (1 g tissue: 5–10 mL buffer), followed by centrifugation at 8000× *g* at 4 °C for 10 min to collect the supernatant. Reaction mixtures were prepared by combining samples, standards, and reagents according to the kit protocol, incubated at 25 °C for 10 min, treated with Reagent 2, and incubated at room temperature for an additional 10 min. Absorbance was measured at 405 nm.

### 4.13. O_2_·^−^ Content Assay

O_2_·^−^ content was determined using a modified protocol based on prior literature [65]. O_2_·^−^ was quantified by its reaction with hydroxylamine to form nitrite (NO_2_^−^), which subsequently reacted with sulfanilic acid and α-naphthylamine to generate a pink azo dye. Mix 0.05 mL of 0.05 M phosphate-buffered saline (PBS, pH 7.8) with 0.1 mL of 10 mM hydroxylamine chloride and incubate at 25 °C for 10 min. Add 0.5 mL of crude enzyme extract (prepared by homogenizing 0.5 g of leaf tissue in 3 mL of 0.05 M PBS, pH 7.8, on ice, followed by centrifugation at 12,000× *g*, 4 °C for 20 min) and incubate at 25 °C for 20 min. Sequentially add 1 mL of 58 mM sulfanilic acid and 1 mL of 7 mM α-naphthylamine, mix, and incubate at 25 °C for 20 min. Add 3 mL chloroform, vortex, and centrifuge at 10,000× *g* for 3 min. Measure the absorbance of the pink aqueous phase at 530 nm.

### 4.14. MDA Content Assay

Malondialdehyde (MDA) content was quantified using a modified protocol based on prior literature, involving its reaction with thiobarbituric acid (TBA) [66]. Briefly, 375 µL of crude enzyme extract (identical to that used for O_2_·^−^ determination) was mixed with 1.25 mL of 20% trichloroacetic acid (TCA) containing 0.5% TBA. The mixture was boiled for 10 min, rapidly cooled, and centrifuged at 1800× *g* for 10 min. Absorbance of the supernatant was measured at 450 nm, 532 nm, and 600 nm to calculate MDA content.

### 4.15. H_2_O_2_ Content Assay

Hydrogen Peroxide (H_2_O_2_) content was determined using a commercial kit (Solarbio Technology Co., Ltd., Beijing, China) following the manufacturer’s protocol. Reagents included Reagent 1 (acetone, self-prepared), Reagent 2 (powder, dissolved in 3 mL concentrated HCl immediately before use), Reagent 3 (6 mL liquid), Reagent 4 (30 mL liquid), and a 1 M H_2_O_2_ standard solution. All reagents were stored at 4 °C. After appropriate pretreatment, samples were combined with reagents, centrifuged, and the supernatant collected. In an EP tube, samples/standards were sequentially mixed with Reagents 1–4, centrifuged, and the precipitate dissolved. Absorbance was measured at 415 nm using a spectrophotometer or microplate reader. H_2_O_2_ content was calculated using ΔA values (ΔA_sample = A_sample − A_blank; ΔA_standard = A_standard − A_blank) according to the provided formula.

### 4.16. Statistical Analysis

After assessing data normality and homogeneity, the statistical method was chosen according to the data distribution characteristics. For data conforming to normal distribution and homogeneity of variance, Analysis of Variance (ANOVA) was employed, followed by Tukey’s test for multiple comparisons. In contrast, when normality or homogeneity assumptions were violated, the Kruskal–Wallis H Test was conducted, with the subsequent Dunn’s Test for assessing statistical significance among groups. Post hoc *p*-values from multiple comparisons underwent Benjamini–Hochberg correction, and the results were labeled using letters (a > b) for identification.

## 5. Conclusions

In this study, through integrative metabolomic and proteomic analyses, we identified key metabolites and proteins involved in MT-mediated alleviation of salt stress in maize seedlings and localized the underlying mechanisms to photosynthesis and antioxidant defense. Salt stress disrupted the redox balance and metabolic processes in maize seedlings, leading to excessive accumulation of ROS and subsequent cellular oxidative damage. Exogenous MT treatment significantly enhanced antioxidant enzyme activities under NaCl stress, thereby mitigating oxidative damage. Additionally, we found that the decline in net photosynthetic rate under salt stress was primarily attributed to photosystem damage rather than stomatal limitations, manifesting as alterations in thylakoid structure and reductions in chlorophyll content, ultimately impairing PSII function. These damages were notably alleviated by MT application. Our findings provide novel insights into the mechanisms of MT-mediated salt stress alleviation, offering theoretical foundations and innovative strategies for breeding salt-tolerant crops and developing eco-friendly stress-resistant products, which are crucial for sustaining agricultural productivity in saline soils.

## Figures and Tables

**Table 1:** Chlorophyll a fluorescence parameters in leaves of maize seedlings under normal cultivation, MT treatment, NaCl treatment, and NaCl_MT treatment conditions.

Parameters	CK	MT	NaCl	NaCl_MT
Fv/Fm	0.800 ± 0.00637 (a)	0.804 ± 0.00615 (a)	0.766 ± 0.00854 (c)	0.785 ± 0.00751 (b)
Fv/Fo	3.99 ± 0.162 (a)	4.02 ± 0.142 (a)	3.27 ± 0.191 (c)	3.60 ± 0.185 (b)
φEo	0.431 ± 0.0142 (a)	0.446 ± 0.0113 (a)	0.347 ± 0.0176 (c)	0.386 ± 0.0149 (b)
ψEo	0.535 ± 0.0174 (a)	0.524 ± 0.0118 (a)	0.431 ± 0.0179 (c)	0.482 ± 0.0110 (b)
Vj	0.466 ± 0.0234 (b)	0.474 ± 0.0129 (b)	0.558 ± 0.0217 (a)	0.495 ± 0.0202 (b)
Vi	0.826 ± 0.0175 (b)	0.833 ± 0.0106 (b)	0.893 ± 0.0299 (a)	0.850 ± 0.0208 (b)
ABS/RC	2.78 ± 0.122 (b)	2.92 ± 0.237 (b)	3.71 ± 0.216 (a)	3.02 ± 0.141 (b)
DIo/RC	0.558 ± 0.0239 (c)	0.569 ± 0.0438 (bc)	0.852 ± 0.0306 (a)	0.618 ± 0.0197 (b)
TRo/RC	2.22 ± 0.112 (b)	2.31 ± 0.199 (b)	2.81 ± 0.158 (a)	2.40 ± 0.124 (b)
ETo/RC	1.19 ± 0.0686 (a)	1.20 ± 0.111 (a)	1.26 ± 0.0769 (a)	1.21 ± 0.0435 (a)

Note: Data are presented as mean ± standard deviation (sd). Each group had 5 biological replicates. Statistical methods employed were ANOVA followed by Tukey’s test or the Kruskal–Wallis H Test with Dunn’s Test, depending on the data distribution. Letters in parentheses denote significant differences (a > b > c).

## Data Availability

The raw metabolomics data have been submitted to the National Genomics Data Center (https://ngdc.cncb.ac.cn/) under the accession number PRJCA047219. The raw proteomics data have been deposited in iProX (https://www.iprox.cn/) with the project ID IPX0013680000.

## Supplementary Materials

The following supporting information can be downloaded at: https://www.mdpi.com/article/10.3390/plants14203129/s1. Supplementary data associated with this article can be found in the word file. Text S1: Identifying the optimal concentrations of NaCl and MT. Figure S1: (A) Plant height and (B) plant dry weight measured after different concentrations of NaCl treatment. Figure S2: (A) Plant height and (B) plant dry weight measured after 100 mM NaCl combined with different concentrations of MT treatment. Figure S3: A schematic summary of the JIP-test (modified after Tsimilli-Michael and Strasser). The energy fluxes and energy bifurcations (wide arrows) represent the outfluxes for energy conservation (gray arrows) and the outfluxes for dissipation (white arrows), respectively. The efficiencies/yields (line arrows) are also shown and further linked with fluorescence signals. For further details, see Table S1. Figure S4: Plant phenotypic images under normal cultivation, MT treatment, NaCl treatment, and NaCl_MT treatment conditions. Figure S5: Basic overview of proteomics. (A) PCA analysis of normalized abundances across groups. (B) Clustering heatmap of normalized abundances across groups. Figure S6: Integrated analysis of metabolomics and proteomics data. (A) Pearson correlation network map of significantly differential metabolites and proteins. (B) Subnetwork with the highest score extracted from the network by MDOCE. Yellow represents metabolites and green represents proteins. Figure S7: Water-use efficiency (WUE) and instantaneous water-use efficiency (iWUE) under normal cultivation, MT treatment, NaCl treatment, and NaCl_MT treatment. Data are presented as mean ± standard deviation (sd). Each group had 5 biological replicates. Statistical methods employed were ANOVA followed by Tukey’s test or Kruskal-Wallis H Test with Dunn’s Test, depending on the data distribution. Letters in parentheses denote significant differences (a > b). (A) CK, (B) MT, (C) NaCl, (D) NaCl_MT. C stands for chloroplast. Figure S8: Ultrastructural images of chloroplasts under 10-micrometer magnification. (A) CK, (B) MT, (C) NaCl, (D) NaCl_MT. C stands for chloroplast. Figure S9: Chlorophyll extraction status across various treatment groups. Figure S10: DAB (A) and NBT (B) staining results of leaf sections. The scale bar in the figures is 1 cm. Table S1: Summary of parameters, formulae and their description using data extracted from chlorophyll fluorescence transients OJIP. Subscript ‘0’ indicates that the parameter refers to the onset of illumination, when all RCs are assumed to be open. Table S2: Summary of identified metabolites.

## Abbreviations

The following abbreviations are used in this manuscript:

DAB	3,3′-diaminobenzidine
ANOVA	analysis of variance
BH	Benjamini–Hochberg
CAT	catalase
Ca	chlorophyll a
Cb	chlorophyll b
DEMs	differentially expressed metabolites
DEPs	differentially expressed proteins
FC	fold change
MDA	malondialdehyde
MT	melatonin
NBT	nitroblue tetrazolium
PPP	pentose phosphate pathway
POD	peroxidase
PS	photosystem
PCA	principal component analysis
ROS	reactive oxygen species
SAH	S-Adenosylhomocysteine
SOD	superoxide dismutase
TBA	thiobarbituric acid
TCA	trichloroacetic acid
TEM	transmission electron microscope
