# Supplementary material for: Elucidating the Mechanistic Role of Exogenous Melatonin in Salt Stress Tolerance of Maize (Zea mays L.) Seedlings: An Integrated Physiological, Metabolomic, and Proteomic Profiling Analysis
Source: Plants (Basel). 2025 Oct 10;14(20):3129. doi: 10.3390/plants14203129 (PMC12566909; doi:10.3390/plants14203129)
Supplement: Supplementary file 1 [file plants-14-03129-s001.zip › Melatonin_Salt_Stress_SI_revised.pdf]

# Supplementary Information

# Elucidating the Mechanistic Role of Exogenous Melatonin in Salt Stress Tolerance of Maize Seedlings: An Integrated Physiological, Metabolomic, and Proteomic Profiling Analysis

Zhichao Wang [#], Linhao Zong [#], Qiqi Cai, Yinjie Fu, Zhiping Gao*, Guoxiang Chen*

Jiangsu Key Laboratory for Biodiversity and Biotechnology, College of Life Sciences, Nanjing Normal University, 1 Wenyuan Rd., Nanjing, Jiangsu, 210023, China

*Authors for correspondence:

Zhiping Gao, Ph.D. Prof. E-mail: 08295@njnu.edu.cn

Guoxiang Chen, Ph.D. Prof. E-mail: gxchen_njnu@163.com

[#] Zhichao Wang and Linhao Zong have contributed equally to this work.

**Text S1: Identifying the optimal concentrations of NaCl and MT**

To screen for the optimal NaCl concentration, five gradient treatments were established by supplementing a half-strength Hoagland nutrient solution with varying NaCl concentrations: 0 mM, 50 mM, 100 mM, 150 mM, and 200 mM. To maintain a consistent nutrient volume, fresh half-strength Hoagland solution was added daily, and the entire solution was replaced with fresh solution of the same composition every two days. After four days of treatment, plant height and biomass were measured (Figure S1). The NaCl concentration used in subsequent experiments was determined based on the ability to induce a notably significant phenotypic difference, specifically 100 mM.

To determine the optimal melatonin (MT) concentration, a half-strength Hoagland nutrient solution was first supplemented with NaCl at the previously identified optimal concentration. Subsequently, five MT concentration gradients were established by adding MT to this solution: 0 μM, 0.1 μM, 1 μM, 10 μM, and 100 μM. To maintain a consistent nutrient volume, fresh half-strength Hoagland solution was added daily, and the entire solution was replaced with fresh solution of the same composition every two days. After four days of treatment, plant height and biomass were measured (Figure S2). The MT concentration employed in subsequent experiments was selected as the one most effectively mitigating stress effects, specifically 1 μM.

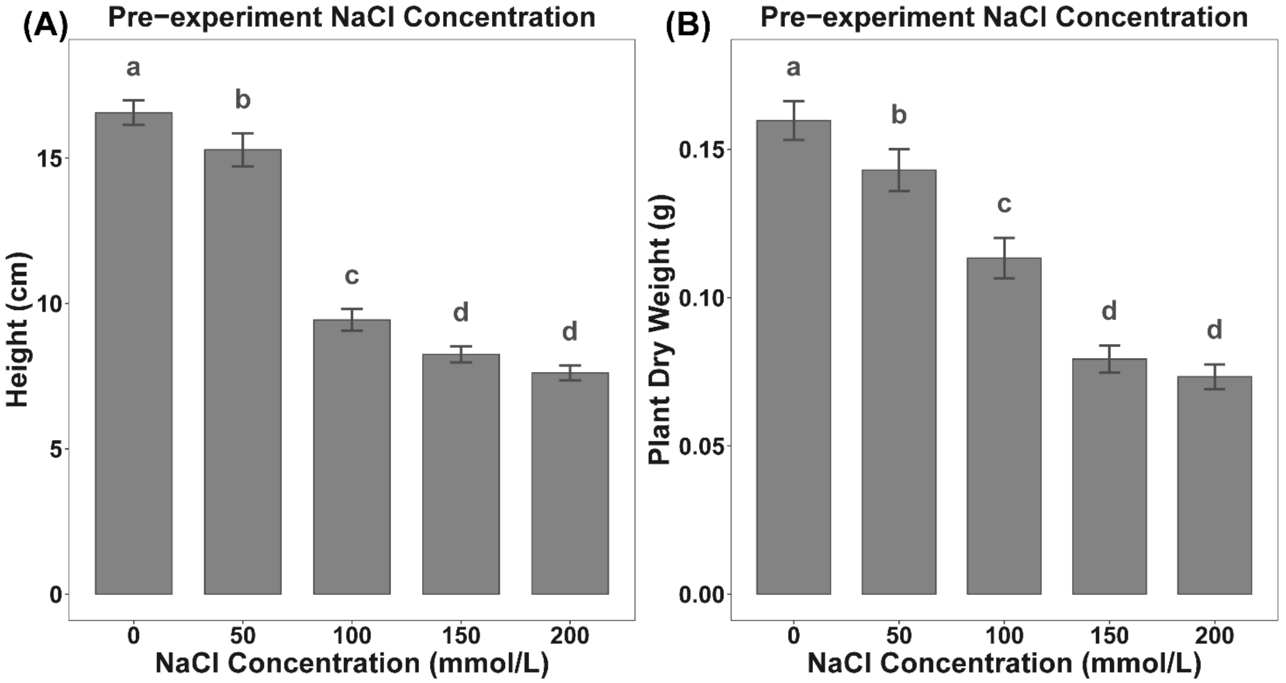


**Figure S1**. (A) Plant height and (B) plant dry weight measured after different concentrations of NaCl treatment.

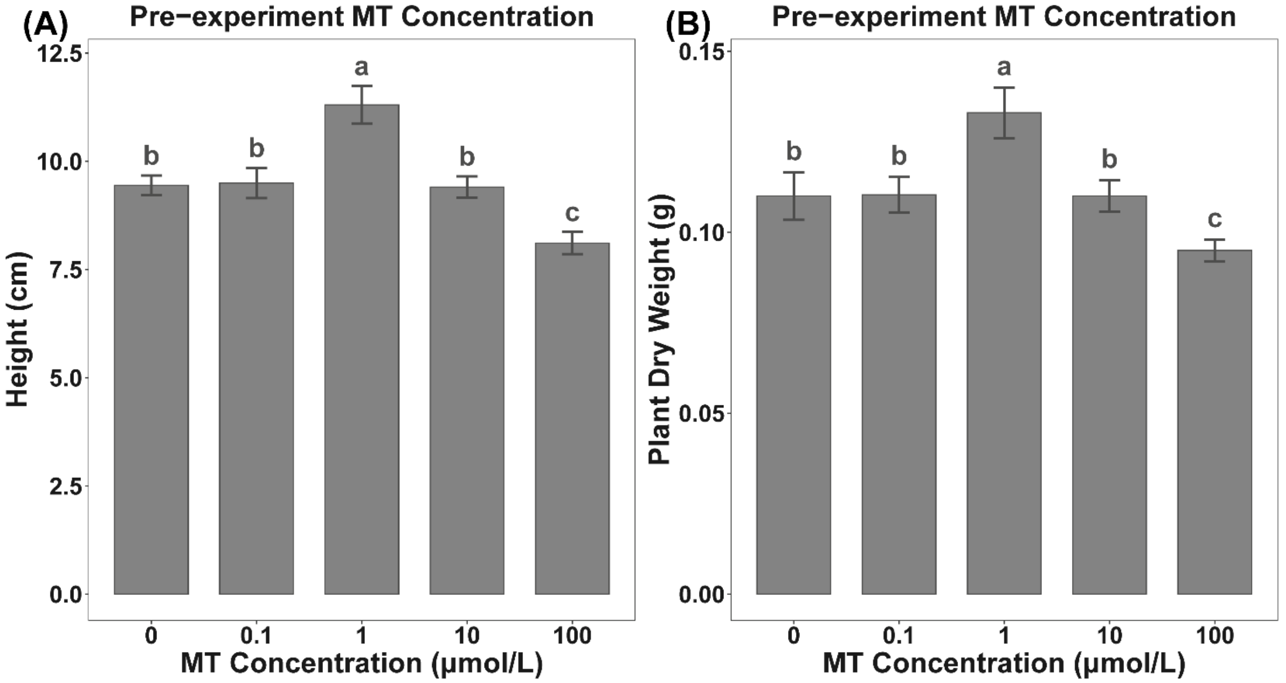


**Figure S2**. (A) Plant height and (B) plant dry weight measured after 100 mM NaCl combined with different concentrations of MT treatment.

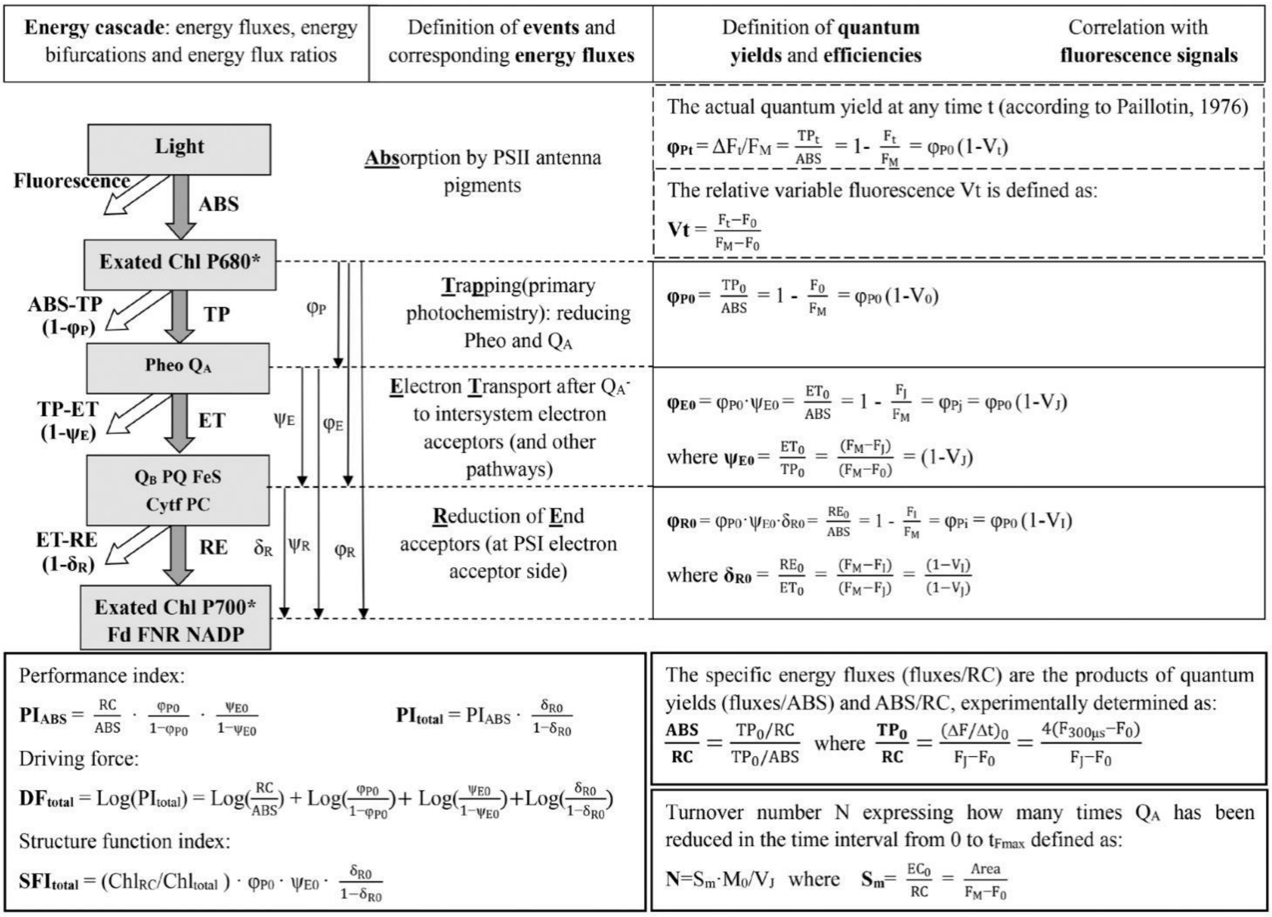


**Figure S3.** A schematic summary of the JIP-test (modified after Tsimilli-Michael and Strasser 2008). The energy fluxes and energy bifurcations (wide arrows) represent the outfluxes for energy conservation (grey arrows) and the outfluxes for dissipation (white arrows), respectively. The efficiencies/yields (line arrows) are also shown and further linked with fluorescence signals. For further details, see Table S1.

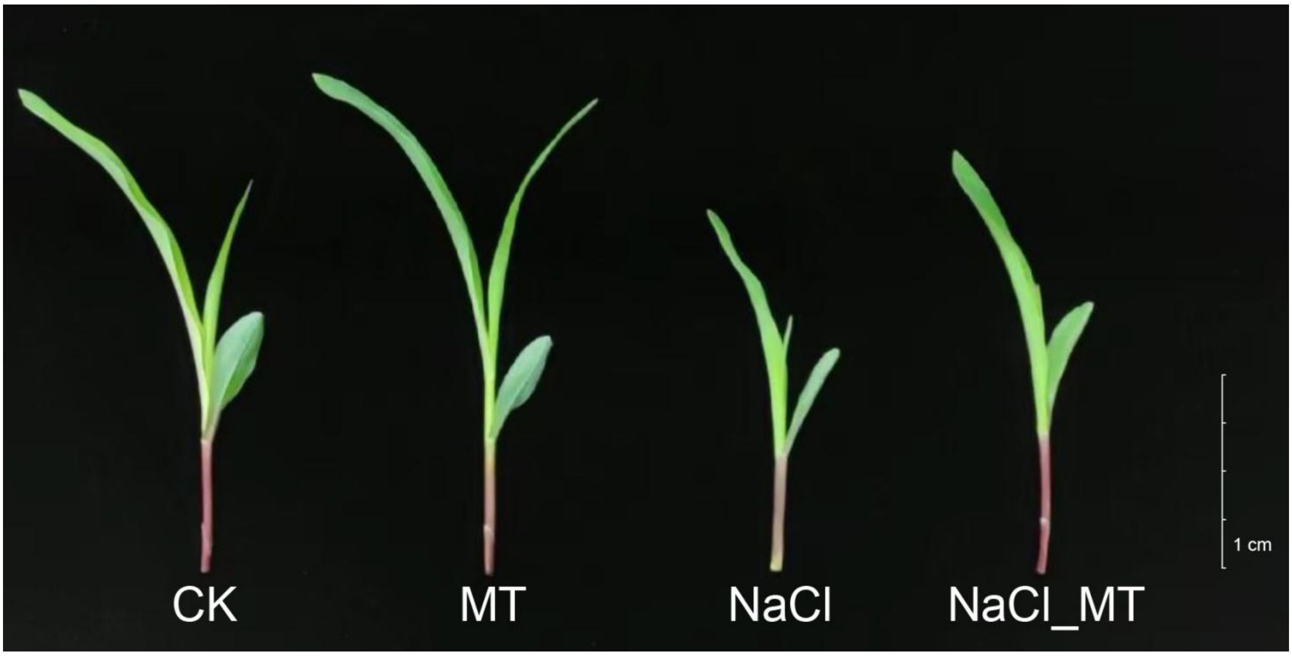


**Figure S4**. Plant phenotypic images under normal cultivation, MT treatment, NaCl treatment, and NaCl_MT treatment conditions.

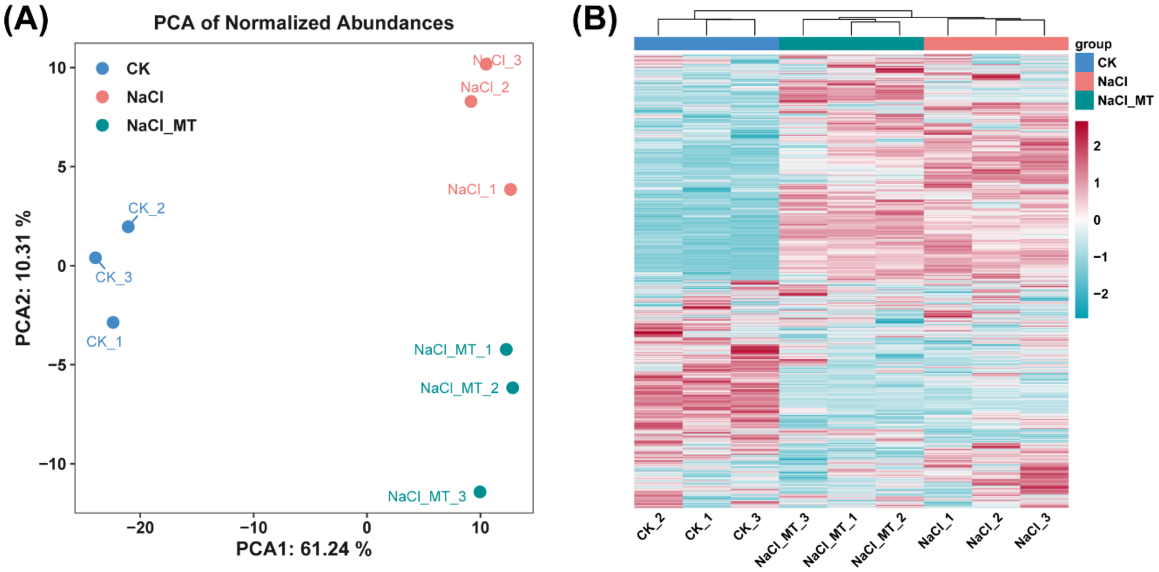


**Figure S5**. Basic overview of proteomics. (A) PCA analysis of normalized abundances across groups. (B) Clustering heatmap of normalized abundances across groups.

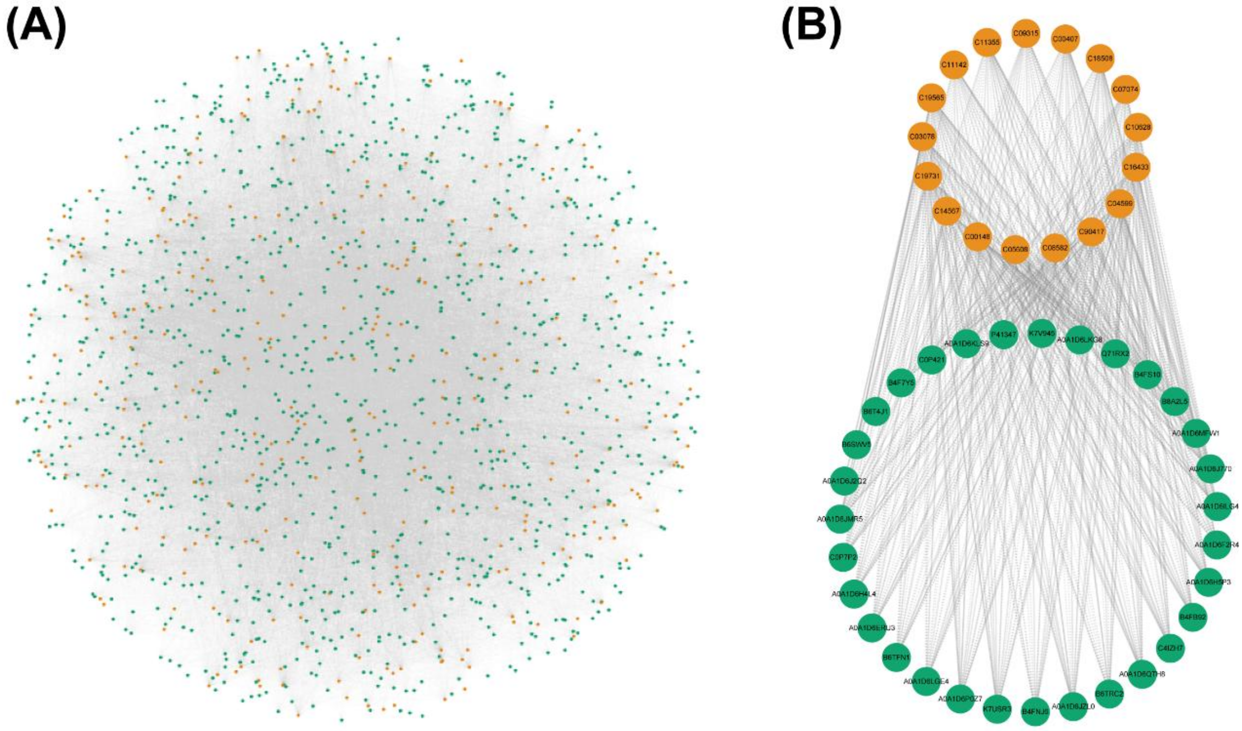


**Figure S6**. Integrated analysis of metabolomics and proteomics data. (A) Pearson correlation network map of significantly differential metabolites and proteins. (B) Subnetwork with the highest score extracted from the network by MDOCE. Yellow represents metabolites and green represents proteins.

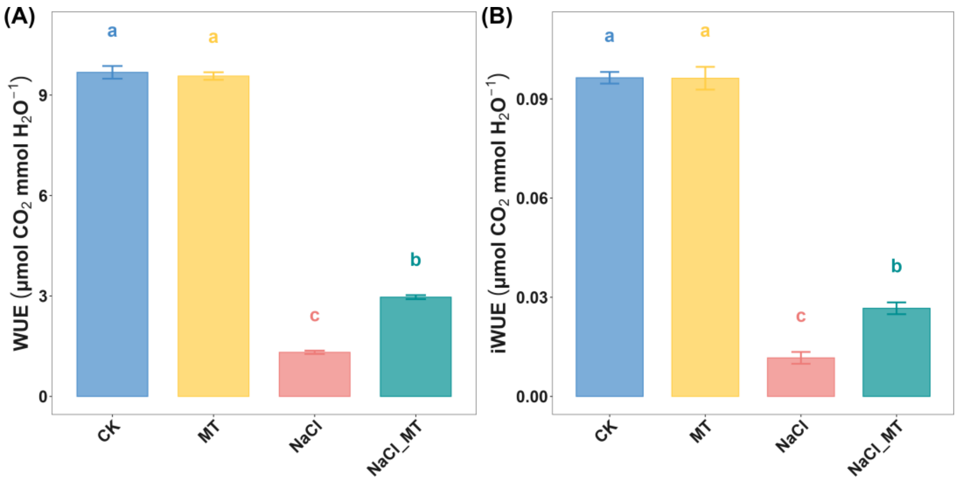


**Figure S7**. Water-use efficiency (WUE) and instantaneous water-use efficiency (iWUE) under normal cultivation, MT treatment, NaCl treatment, and NaCl_MT treatment. Data are presented as mean ± standard deviation (sd). Each group had 5 biological replicates. Statistical methods employed

were ANOVA followed by Tukey's test or Kruskal-Wallis H Test with Dunn's Test, depending on the data distribution. Letters in parentheses denote significant differences (a > b).

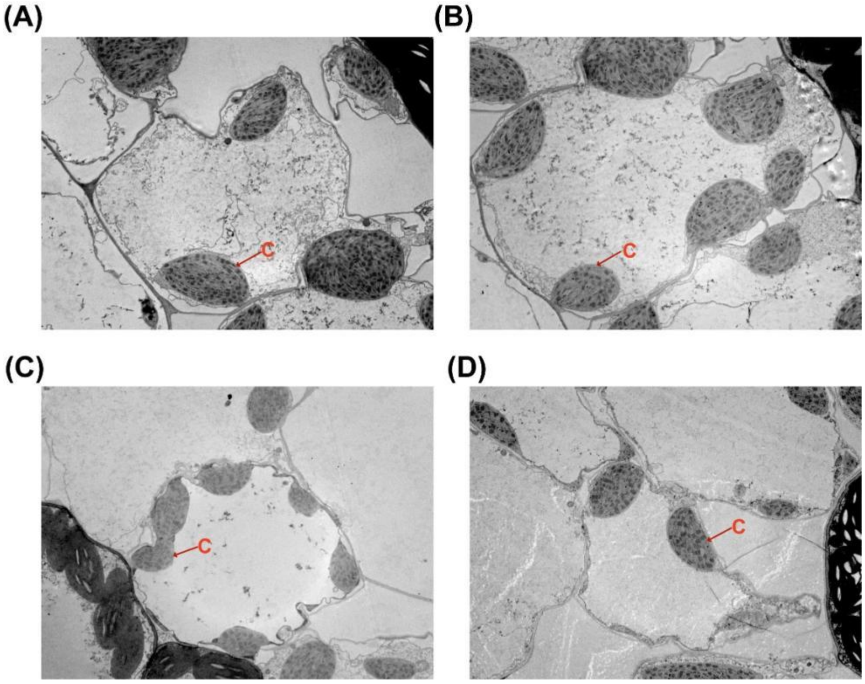


**Figure S8**. Ultrastructural images of chloroplasts under 10-micrometer magnification. (A) CK, (B) MT, (C) NaCl, (D) NaCl_MT. C stands for chloroplast.

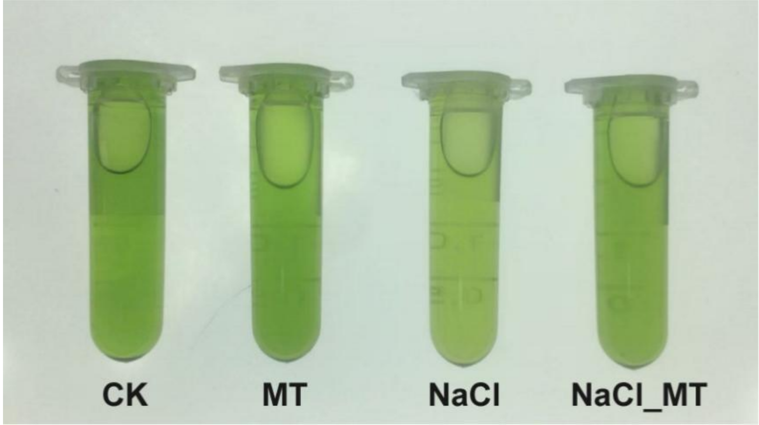


**Figure S9**. Chlorophyll extraction status across various treatment groups.

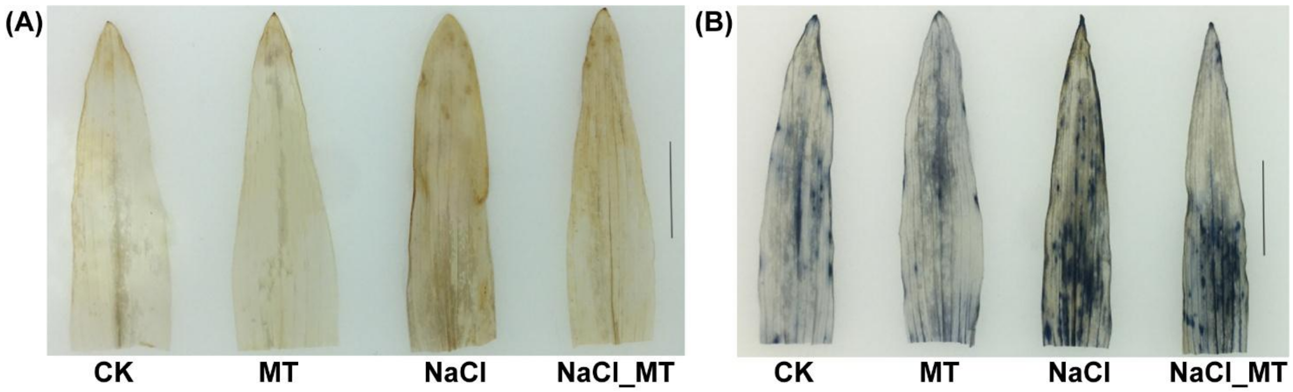


**Figure S10**. DAB (A) and NBT (B) staining results of leaf sections. The scale bar in the figures is 1 cm.

**Table S1. Summary of parameters, formulae and their description using data extracted from chlorophyll fluorescence transients OJIP. Subscript '0' indicates that the parameter refers to the onset of illumination, when all RCs are assumed to be open.**

| Fluorescence parameters | Description |
|---|---|
| Selected OJIP parameters | |
| $F_t$ | Fluorescence at time t after onset of actinic illumination |
| $F_0$ | Minimum fluorescence, when all PSII RCs are closed |
| $F_J = F_{2\ ms}$ | Fluorescence intensity at the J-step (2 ms) of OJIP |
| $F_I = F_{30\ ms}$ | Fluorescence intensity at the I-step (30 ms) of OJIP |
| $F_M = F_P$ | Maximum fluorescence, when all PSII RCs are closed |
| $Vt = (F_t - F_0)/(F_M - F_0)$ | Relative variable fluorescence at time t |
| $V_J = (F_{2\ ms} - F_0)/(F_M - F_0)$ | Relative variable fluorescence at the J-step (2 ms) |
| $V_I = (F_{30\ ms} - F_0)/(F_M - F_0)$ | Relative variable fluorescence at the I-step (30 ms) |
| $Area = {}^{tFmax}{}_0\int(F_M - F_t)dt$ | Total complementary area between the fluorescence induction curve and $F = F_M$ |
| $M_0 = 4\ (F_{300\ \mu s} - F_0)/(F_M - F_0)$ | Approximated initial slope (in $ms^{-1}$) of the fluorescence transient $V = f_t$ |

| | |
|---|---|
| $S_m = EC_0/RC = Area/(F_M - F_0)$ | Working integral of the energy needed to close all reaction centers |
| $N = S_m \times M_0/V_J$ | Times that $Q_A$ has been reduced in the time interval from 0 to $t_{Fmax}$ |
| $S_m/t_{Fmax}=[RC_{open}/(RC_{close} + RC_{open}]av=[Q_A/Q_{A(total)}]av$ | The average (av) redox state of $Q_A^-/Q_A$ in the time interval from 0 to $t_{Fmax}$, or the average electron transport activity or the average fraction of open RCs. |

Specific fluxes or activities expressed per reaction center (RC)

| | |
|---|---|
| $ABS/RC = (M_0/V_J)/[1 - (F_0/F_M)]$ | Absorption flux per RC |
| $TR_0/RC = (M_0/V_J)$ | Trapped (maximum) energy flux (leading to $Q_A$ reduction) per RC at t = 0 |
| $ET_0/RC = (M_0/V_J) \times (1 - V_J)$ | Maximum electron transport flux (further than $Q_A^-$) per RC at t = 0 |

Yields or flux ratios

| | |
|---|---|
| $\varphi_{P0} = TR_0/ABS = F_V/F_M = [1 - (F_0/F_M)]$ | Maximum quantum yield of primary photochemistry |
| $\varphi_{E0} = ET_0/ABS = [1 - (F_0/F_M)] \times (1 - V_J)$ | Quantum yield of electron transport |
| $\psi_0 = ET_0/TR_0 = 1 - V_J$ | Probability (at time 0) that a trapped exciton moves an electron into the electron transport chain beyond $Q_A^-$ |
| $\delta_{R0} = RE_0/ET_0 = (1 - V_I)/(1 - V_J)$ | Efficiency with which an electron can move from the reduced intersystem electron acceptors to the PSI end electron acceptors |

| | |
|---|---|
| $\varphi_{R0} = RE_0/ABS = \varphi_{P0} \times \varphi_{E0} \times \delta_{Ro}$ | Quantum yield for the reduction of end acceptors of PSI per photon absorbed |
| Phenomenological fluxes or activities expressed per excited cross section (CS) | |
| $ABS/CS_m = F_M$ | Absorption flux of photons per cross section, approximated by $F_M$ |
| $TR_0/CS_m = \varphi_{P0} \times (ABS/CS_m)$ | Phenomenological fluxes for trapping per cross section, approximated by $F_M$ |
| $ET_0/CS_m = \varphi_{E0} \times (ABS/CS_m)$ | Potential electron transport per cross section, approximated by $F_M$ |
| $DI_0/CS_m = (ABS/CS_m) – (TR_0/CS_m)$ | Dissipation per cross section, approximated by $F_M$ |
| Performance index | |
| $PI_{ABS} = (RC/ABS) \times [\varphi_{P0}/(1 – \varphi_{P0})] \times [\psi_0/(1 – \psi_0)]$ | Performance index (potential) for energy conservation from exciton to the reduction of intersystem electron acceptors |
| $PI_{total} = PI_{ABS} \times [\delta_{R0}/(1 – \delta_{R0})]$ | Performance index (potential) for energy conservation from exciton to the reduction of PSI end acceptors |
| Driving force | |
| $DF_{total} = \log(PI_{total}) = \log(PI_{ABS})+\log[\delta_{R0}/(1 – \delta_{R0})]$ | Proton motor force on an absorption basis, created by summing the partial driving forces for each of the several energy bifurcations |
| Structure function index | |
| $SFI_{total} = (Chl_{RC}/Chl_{total}) \times \varphi_{P0} \times \psi_0 \times [\delta_{R0}/(1 – \delta_{R0})]$ | Structure function index combining structural and functional criteria of PSII |
